# Sex Workers Should not Be Forgotten in Africa’s COVID-19 Response

**DOI:** 10.4269/ajtmh.20-1045

**Published:** 2020-09-15

**Authors:** Yusuff Adebayo Adebisi, Aishat Jumoke Alaran, Rafiat Tolulope Akinokun, Alumuku Iordepuun Micheal, Esther Bosede Ilesanmi, Don Eliseo Lucero-Prisno

**Affiliations:** 1 Faculty of Pharmacy, University of Ibadan, Ibadan, Nigeria;; 2 Faculty of Pharmaceutical Sciences, University of Ilorin, Ilorin, Nigeria;; 3 Faculty of Nursing Science, Ladoke Akintola University of Technology, Ogbomoso, Nigeria;; 4 School of Medicine, V. N. Karazin Kharkiv National University, Kharkiv, Ukraine;; 5 Department of Global Health and Development, London School of Hygiene and Tropical Medicine, London, United Kingdom

## Abstract

COVID-19 is a global health emergency facing many countries around the world. Sex workers in Africa are among one of the vulnerable populations disproportionately affected by the COVID-19 pandemic on the continent. Sex workers are excluded from African government safety net, and this may force some sex workers back to sex work amid the COVID-19 pandemic. Because of the nature of sex work, physical distancing and other precautionary measures are impossible to observe, further compromising COVID-19 response. Sex workers in Africa have been known to face high levels of stigma and discrimination, including limited access to healthcare services. Disruption in HIV care and prevention services due to the pandemic among this key population may have negative impacts on the hard-won achievements in HIV response in Africa. In addition, stigma and discrimination toward sex workers could also make contact tracing challenging and limit access to COVID-19 testing among this vulnerable group. With the adoption of the 2030 Agenda for the UN Development Program, UN member states all pledged to ensure “no one will be left behind” and to “endeavor to reach the furthest behind first.” This could not be more important than now as sex workers as a part of the population are left behind in COVID-19 response in Africa. It is important that the African government should ensure collective and inclusive response in the fight against COVID-19. Sex workers should not be forgotten in Africa’s COVID-19 response because no one is safe, until all are safe.

Since the emergence of the COVID-19 pandemic, countries all over the world had put up measures aimed at protecting their populace and reducing the spread of the virus. As countries adjust their public health measures, it is imperative that the most vulnerable members of the society are protected and supported.^1^ Sex workers belong to the vulnerable and key populations in Africa who are highly stigmatized, criminalized, and marginalized.^2^ Sex work in Africa has always been surrounded by controversies and debates concerning its cultural, legal, and social frameworks. With the stigma and criminalization of the trade in Africa, sex workers have often had their rights infringed upon, including limited access to healthcare services and legal services.^3,4^ Measure Evaluation^5^ reported that social stigma, gender-based violence, and discriminatory policies including criminalization of all lifestyles of key populations hinder their access to care and treatment. These groups are hard to reach with health interventions and behavioral change interventions because they are often mobile and “hidden.”^5^ In the face of the current COVID-19 pandemic, this at-risk group is made even more vulnerable and further pushed to the farthest margins as it affects this group disproportionately.^6^ It is also worrisome that as the economic threat of COVID-19 continues to increase and the population are pushed to more difficulties, there could be a surge of sex workers, further posing challenges to COVID-19 containment efforts.

Sex workers in Africa are among the communities suffering the most because of the COVID-19 pandemic, as lockdown and police crackdowns leave millions without income.^7^ They are being forgotten in government responses to the COVID-19 crisis and are finding themselves unable to provide for themselves and their families, thereby increasing their vulnerability.^6^ In addition, sex workers are also facing harsh business conditions such as low pay for their services, as the number of clients becomes very low.^8,9^ Because of relative collapse of their industry, some sex workers are unable to afford rent or alternative forms of shelter, making them further vulnerable to harassment and exploitation.^10^ Sex workers do not also have access to employment insurance or many of the recent governments’ emergency support and rent assistance for citizens,^11^ and because of the cultural criminalization of sex work in Africa, they are not entitled to various COVID-19 social services and safety net. They are also often denied help, further reiterating poverty, inequality, and marginalization among this group.^12^ For instance, governments in Botswana and South Africa provided relief packages to most of their citizens, sex workers were mostly excluded, and, as such, nongovernmental organizations had to step up to render assistance to this population.^13^ Sex workers in Nigeria and Uganda have also reported on how exclusion from government safety net has forced them back to sex work amid COVID-19.^8^ Because of the nature of sex work, physical distancing and other precautionary measures are impossible to observe, further compromising COVID-19 response.

Sex workers in Africa are often exposed to violence, rape, and all sorts of physical abuse. Amid the COVID-19 pandemic, there has also been a spike in violence faced by sex workers, from clients, police, and even community members who blame them for spreading the disease.^2^ With criminalization of sex work in Africa, sex workers are therefore more exposed to punitive measures to enforce COVID-19 regulations. Increased policing and curfew can expose them to more violence, abuse, and harassment.^6^ Furthermore, sex workers have always been at a higher risk of contracting and spreading infectious disease such as HIV. Many studies have noted that HIV prevalence among sex workers is more than 10- to 20-fold higher than that in the general population.^3,4^ However, in recent times, empowerment and education of sex workers have been proven to help curb the spread of HIV.^14^ Availability of antiretroviral treatment to those living with HIV and education on safe sex practices such as condom use to reduce the transmission of sexually transmitted illnesses have gone a long way to reduce the prevalence of the disease.^15^ COVID-19 threatens the achievements made as access to health services and HIV treatment and prevention services are made difficult because of the pandemic. Many sex workers complain of limited access to health services as well as dwindled condom supply.^16^ It is therefore not impossible that the hard-won achievements in HIV response in Africa could be disrupted by the COVID-19 pandemic. More so, mental health problems among African sex workers are likely to also be ensued from anxiety over income, food, and housing, alongside concerns about infection and danger from continuing to work in the absence of sexual and social protection.^17^ They are becoming increasingly worried about their health,^18^ and those living with HIV are greatly concerned that their prior condition, with its negative effects on their immune system, will make them especially vulnerable to contracting COVID-19 and may worsen their health.^10^

With the adoption of the 2030 Agenda for the UN Development Program, UN member states all pledged to ensure “no one will be left behind” and to “endeavor to reach the furthest behind first.”^19^ This could not be more important than now as sex workers as a part of the population are left behind in COVID-19 response in Africa. The framework proposed three approaches to “*examine* the disadvantages people face, to *empower* those who are left behind, and to *enact* inclusive far-sighted and progressive sustainable development goals.”^19^ It is also of point to note that there is no universal health coverage without every aspect of the population being reached and that includes even those in the farthest margins. Therefore, it is essential that for African states to achieve COVID-19 control and healthy communities, all aspects of the population must be covered, including the vulnerable groups. As Winnie Byanyima, the executive director of The Joint United Nations Programme on HIV/AIDS (UNAIDS), stated: “Human rights law mandates that human rights are inalienable, universal, interdependent, and indivisible,” therefore “ensuring that this is a reality for all—especially the most vulnerable among us—is not only essential during this pandemic but will also build the resilient communities we need to emerge from it.”^6^ Efforts need to be made by governments across Africa toward decriminalization of sex work, continuing education, and empowerment of sex workers. Support from the African governments even during this pandemic period will go a long way toward sex workers’ integration and inclusion into the society.

Involving communities in social protection schemes, health services, and information dissemination will enable sex workers and other key populations to protect their health during this pandemic.^20^ The WHO^21^ reported that eliminating sexual violence against sex workers could lead to 20% reduction in new HIV infection. Therefore, access to HIV treatment and prevention or to vital services addressing domestic or other forms of violence is crucial.^21^ High levels of stigma and discrimination toward sex workers could also make contact tracing challenging and limit access to COVID-19 testing. Stigma remains an important issue for a large proportion of sex workers—when they seek timely professional help, openly disclose their sex work identity, and seek comprehensive healthcare services in Africa.^22^ Addressing stigma is certainly crucial for connecting marginalized sex workers to vital health services, including antiretroviral therapy for those living with HIV.^23^ This becomes imperative amid this ravaging COVID-19 pandemic. Distribution of hand sanitizers, soaps, condoms, and personal protective equipment, including the utilization of person-centered services to address needs associated with mental health, drug use, physical and sexual violence, and sexual and reproductive health, including HIV treatment, remains pertinent among the vulnerable groups including sex workers.^1^ This further reinforces the need for unique COVID-19 response in Africa as previously reported by Lucero-Prisno et al.^24^ It is important that the African government should ensure collective and inclusive response in the fight against COVID-19. Everyone is not safe, until all is safe.
